# Outcomes of CAR-T Cell Therapy Recipients Admitted to the ICU: In Search for a Standard of Care—A Brief Overview and Meta-Analysis of Proportions

**DOI:** 10.3390/jcm12186098

**Published:** 2023-09-21

**Authors:** Catalin Constantinescu, Vlad Moisoiu, Bogdan Tigu, David Kegyes, Ciprian Tomuleasa

**Affiliations:** 1 Department of Hematology, Iuliu Hatieganu University of Medicine and Pharmacy, 400349 Cluj-Napoca, Romania; constantinescu.catalin@ymail.com (C.C.); ciprian.tomuleasa@gmail.com (C.T.); 2 Department of Anesthesia and Intensive Care, Iuliu Hatieganu University of Medicine and Pharmacy, 400349 Cluj-Napoca, Romania; 3 Intensive Care Unit, Emergency Hospital, 400006 Cluj-Napoca, Romania; 4 Medfuture Research Center for Advanced Medicine, Iuliu Hatieganu University of Medicine and Pharmacy, 400337 Cluj-Napoca, Romania; adrianbogdantigu@gmail.com; 5 Department of Neurosurgery, Iuliu Hatieganu University of Medicine and Pharmacy, 400349 Cluj-Napoca, Romania; vlad.moisoiu@gmail.com

**Keywords:** intensive care, critical illness, CAR-T cell, chimeric antigen receptor-T cell, sepsis, hematological malignancies

## Abstract

Objective: Our primary objective was to describe the baseline characteristics, main reasons for intensive care unit (ICU) admission, and interventions required in the ICU across patients who received CAR-T cell immunotherapy. The secondary objectives were to evaluate different outcomes (ICU mortality) across patients admitted to the ICU after having received CAR-T cell therapy. Materials and Methods. We performed a medical literature review, which included MEDLINE, Embase, and Cochrane Library, of studies published from the inception of the databases until 2022. We conducted a systematic review with meta-analyses of proportions of several studies, including CAR-T cell-treated patients who required ICU admission. Outcomes in the meta-analysis were evaluated using the random-effects model. Results: We included four studies and analyzed several outcomes, including baseline characteristics and ICU-related findings. CAR-T cell recipients admitted to the ICU are predominantly males (62% CI-95% (57–66)). Of the total CAR-T cell recipients, 4% CI-95% (3–5) die in the hospital, and 6% CI-95% (4–9) of those admitted to the ICU subsequently die. One of the main reasons for ICU admission is acute kidney injury (AKI) in 15% CI-95% (10–19) of cases and acute respiratory failure in 10% CI-95% (6–13) of cases. Regarding the interventions initiated in the ICU, 18% CI-95% (13–22) of the CAR-T recipients required invasive mechanical ventilation during their ICU stay, 23% CI-95% (16–30) required infusion of vasoactive drugs, and 1% CI-95% (0.1–3) required renal replacement therapy (RRT). 18% CI-95% (13–22) of the initially discharged patients were readmitted to the ICU within 30 days, and the mean length of hospital stay is 22 days CI-95% (19–25). The results paint a current state of matter in CAR-T cell recipients admitted to the ICU. Conclusions: To better understand immunotherapy-related complications from an ICU standpoint, acknowledge the deteriorating patient on the ward, reduce the ICU admission rate, advance ICU care, and improve the outcomes of these patients, a standard of care and research regarding CAR-T cell-based immunotherapies should be created. Studies that are looking from the perspective of intensive care are highly warranted because the available literature regarding this area is scarce.

## 1. Background

Immunotherapy with chimeric antigen receptor (CAR)-T cell is expanding in the field of hematology [1,2,3] and has gained much attention in recent years. Along with the administration of these immunotherapies, complications also arise, the most common ones being cytokine release syndrome (CRS) and immune effector cell-associated neurotoxicity syndrome (ICANS), which, when severe, often require admission and complex management into an intensive care unit (ICU) [4,5,6,7].

The aim of this study was to evaluate the literature experience and perform a meta-analysis of proportions regarding the adult recipients of CAR-T cell immunotherapy who were admitted to the ICU and, based on this, draw several conclusions.

Most studies that describe patients who were treated with CAR-T cell therapy have analyzed the clinical experience from the starting point of lymphodepletion until the very end of treatment but with limited information given regarding the patients who were admitted to the ICU with no mention of the resources used or required life-saving interventions that could have improved their different outcomes. Therefore, the information concerning this segment of practice is scarce [8]. Current ICU practices in CAR-T cell recipients admitted to the ICU are deduced from the general ICU population and are not individualized accordingly.

As intensive care physicians, it is important to acknowledge the currently available information and experiences of the other medical specialties regarding immunotherapies and contribute by creating a standard of care for future ICU patients treated with CAR-T cell therapy. It is mandatory to have know-how regarding the CAR products available, toxicities observed, and available treatment of toxicities, and to update ourselves with the specifics of practices and interventions applicable within the ICU when caring for critically ill CAR patients.

After searching the literature for randomized controlled trials, guidelines, and other available information regarding this segment, we found just four studies [8,9,10,11] addressing this population segment. This underlines the importance of conducting future studies in a systematic manner to help improve the current practice.

## 2. Materials and Methods

This research was planned and undertaken using the recommendations of the Cochrane Collaboration [12] and the preferred reporting items for systematic reviews and meta-analyses (PRISMA) guidelines [13]. Clinical studies evaluating different outcomes like baseline characteristics, hospital and ICU mortality, reasons for ICU admission, and ICU interventions in CAR-T cell recipients admitted to the ICU were identified. Our primary objective was to describe the baseline characteristics, main reasons for ICU admission, and interventions required in the ICU across patients who received CAR-T cell therapy. The secondary objectives were to evaluate different outcomes, like ICU and hospital mortality, across patients admitted to the ICU after having received CAR-T cell therapy.

## 3. Data Sources and Search Strategy

A systematic literature search was undertaken using the medical literature database, which included MEDLINE, Embase, and Cochrane Library, with studies published from the inception of databases until 2022. Keywords and title searches included a combination of: 

(“intensive care”[tw] OR „critical care”[tw] OR “Critical Care Outcomes”[Mesh] OR “Critical Care”[Mesh] OR “Intensive Care Units”[Mesh]) AND (“Chimeric antigen receptor”[tw] OR “Immunotherapy, Adoptive”[Mesh]).

Hand-searching of the references of research papers was also undertaken until no new studies were identified. The search strategy was constructed based on the Populations, Interventions, Comparators, and Outcomes (PICO) structure. Search results were managed via Excel (Microsoft Corporation, Redmond, WA, USA), with duplicate references removed.

## 4. Study Selection

The eligibility for inclusion of research papers in this study was considered independently by two authors (CC and CT). Due to the lack of systematic randomized trials and heterogeneity among the studies, we included all the available studies that addressed patients who were treated with CAR T-cell therapy and were admitted to the ICU. Full texts of the potentially relevant subjects were analyzed, and those who met the eligibility criteria and had at least one outcome measure of interest were included. Eligible studies included published manuscripts that offered information regarding CAR-T recipients who required admission to the ICU for different reasons, either related or unrelated to CAR-T treatment. Review papers, non-adult populations, non-ICU manuscripts that included the same patients used in two different studies [14], and papers that lacked information regarding the ICU period admission were excluded.

## 5. Inclusion and Exclusion Criteria

Eligible patients are aged 18 years or older, have received CAR-T cell therapy in the past 30 days, and have been admitted to intensive care for any reason. The primary endpoints were information regarding different outcomes such as mortality, reasons for ICU admission, ICU interventions, and incidence of CRS and ICANS. In order to be included in the meta-analysis, studies had to meet all the inclusion criteria. 

## 6. Data Extraction and Synthesis

Data extracted from each paper include: the first author’s name and publication year, study design, total participants in the study, sex, patients treated with CAR-T cell therapy, underlying malignancy, type of CAR-T cell treatment, hospital and ICU mortality, reasons for ICU admission, information regarding toxicities of CAR-T cell treatment including CRS and ICANS, SOFA score, therapy instituted in ICU, readmission in ICU, length of hospital and ICU stay.

## 7. Statistical Methods

Statistical analyses were performed using the statistical software R studio (version 2023.03.0+386) and MedCalc software (version 22.013) to conduct meta-analyses of proportions. Regardless of the amount of heterogeneity encountered between the studies, we used the DerSimonian–Laird random-effects model [15] for the meta-analyses, as Cochran’s Q and I^2^ statistic values indicated [16]. Heterogeneity (i.e., variation in outcomes between studies) was assessed based on the significance of the between-study variation using the Chi-square test and I^2^ statistic. Substantial heterogeneity was assumed if the I^2^ value was above 50% [17]. A *p*-value of 0.10, rather than the conventional level of 0.05, was used to determine statistical significance because the interpretation of the chi-squared test has low power in the situation of a meta-analysis with studies that have small sample sizes or are few. If only a median data range was reported, the mean and standard deviation were estimated according to the method provided in the Cochrane Handbook for Systematic Reviewers [17]. Mean values were assumed to be equivalent to the median value, while the standard deviation was assumed to be a quarter of the data range—formula (max − min)/4. 

Thresholds for the interpretation of I^2^ (heterogeneity) [12] are as follows:

0% to 40%: might not be important;

30% to 60%: may represent moderate heterogeneity;

50% to 90%: may represent substantial heterogeneity;

75% to 100%: considerable heterogeneity.

We were not able to perform subgroup analyses due to the few studies available.

## 8. Results

**Figure 1:**
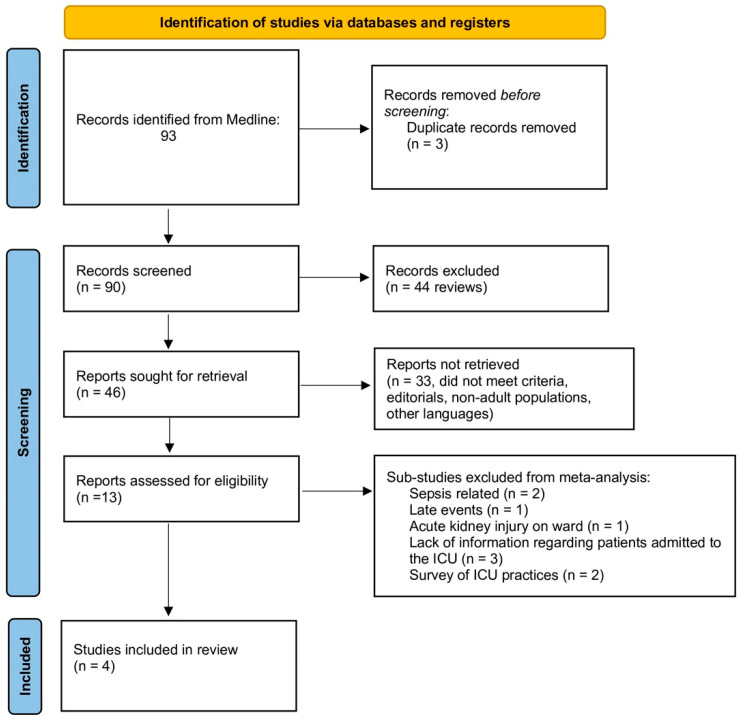
Prisma flow diagram.

The electronic search resulted in 93 potential manuscripts to take into consideration. After the creation of the database and following review of the abstracts or the complete paper when necessary, using the inclusion and exclusion criteria mentioned above, 89 papers were excluded leaving four studies published between 2020–2022, with the characteristics mentioned in Table 1, comprising 379 patients, which however exhibited significant heterogeneity concerning most of the reported parameters, with some of the analyzed variables having fewer patients included (Figure 1). It is worth remembering that several outcomes had high heterogeneity among studies, but we applied a random-effects model of meta-analysis to summarize the results.

**Figure 2:**
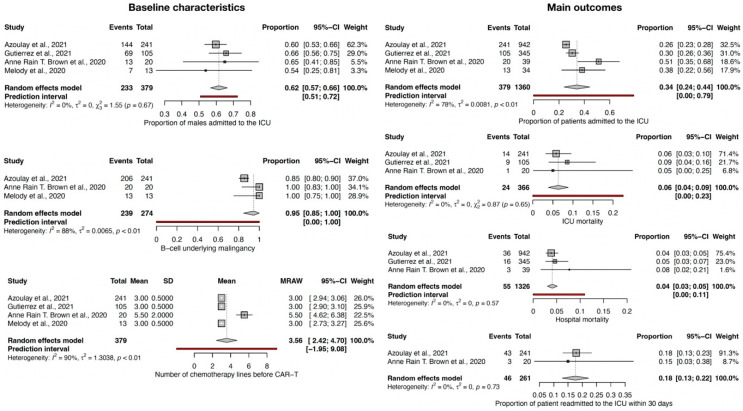
Forest plot for baseline characteristics and main outcomes [8,9,10,11].

The results of the baseline characteristics (Figure 2) showed that CAR-T cell recipients admitted to the ICU are mostly males (62% CI-95% (57–66)). The most predominant malignancy for which these patients were treated with CAR-T therapy is lymphoma (on average 95% CI-95% (85–99)), and these are patients who had to follow multiple lines of chemotherapy before CAR-T cell infusion (on average, the mean was 3.56, CI-95% (2.42; 4.70)). Importantly, this meta-analysis was comprised predominantly of patients with lymphoma, and whether similar figures also apply to other types of hematologic malignancies remains to be established. Considerable heterogeneity (I^2^ over 75% and *p* < 0.10) was present among studies for associated malignancies and the number of chemotherapy lines before CAR-T infusion, but low heterogeneity for the incidence of ICU admission regarding sex, with *p* > 0.10.

From the standpoint of main outcomes (Figure 2), the results showed that 34% CI-95% (24–44) of the total recipients of CAR-T cell require ICU admission, of which, on average, 6% CI-95% (4–9) of those admitted to the ICU subsequently die. Overall hospital mortality was 4% CI-95% (3–5). Moreover, the readmission rate to the ICU within 30 days was 18% CI-95% (13–22). Given the low heterogeneity between studies regarding ICU mortality, hospital mortality, and the need for readmission within 30 days, these are salient features of this population of CAR-T cell recipients. Only the incidence of ICU admission showed a substantial level of heterogeneity among studies (I^2^ of 80%).

**Figure 3:**
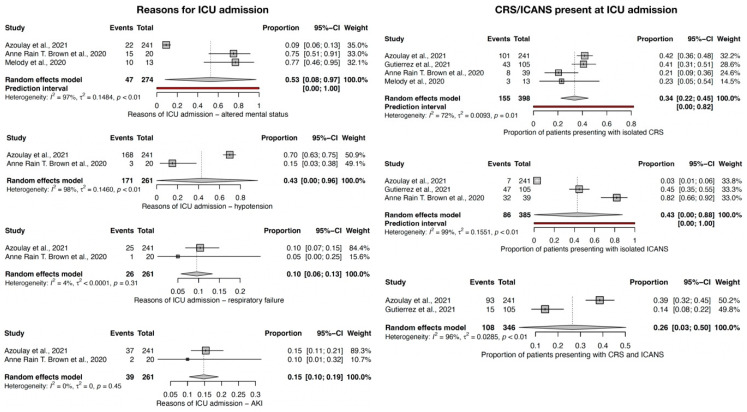
Forest plot for main reasons for ICU admission [8,9,10,11].

Regarding the reasons for ICU admission (Figure 3), 15% CI-95% (10–19) of the CAR-T recipients were admitted to the ICU because of acute kidney injury (AKI), and 10% CI-95% (6–13) were admitted to the ICU because of acute respiratory failure. Both outcomes had a low level of heterogeneity and *p* > 0.10. Hypotension was also present when the patient was admitted to the ICU in 43% CI-95% (1–96) of the CAR-T recipients, and altered mental status was encountered in 53% CI-95% (8–97) of cases, but both showed a considerable degree of heterogeneity among studies (I^2^ > 90%).

For those patients admitted to the ICU, isolated CRS was present in 34% CI-95% (22–45) and isolated ICANS in 43% CI-95% (1–88) of the CAR-T cell recipients. Both were present in 26% CI-95% (3–50) of CAR-T cell recipients. There was a considerable level of heterogeneity reported among studies.

**Figure 4:**
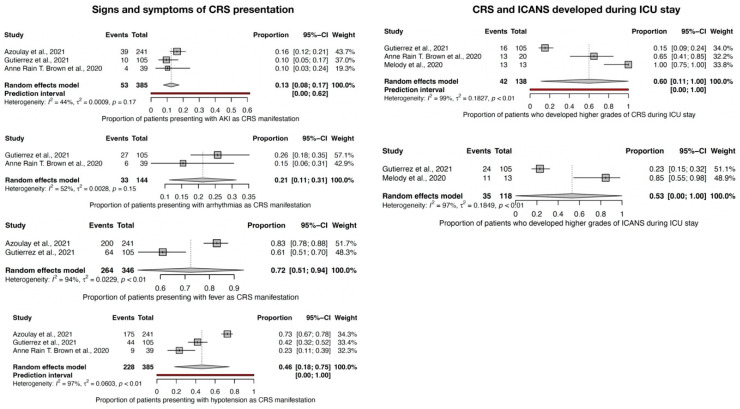
Forest plot for signs and symptoms of CRS presentation and forest plot for higher grades of CRS and ICANS development during ICU stay [8,9,10,11].

From the point of view of the signs and symptoms of CRS presentation (Figure 4), AKI was the main manifestation of CRS presentation in 13% CI-95% (8–17), arrhythmias in 21% CI-95% (11–31), fever in 72% CI-95% (51–94), and hypotension in 46% CI-95% (18–75) of the CAR-T cell recipients. Low heterogeneity and *p* > 0.10 were reported for AKI and arrhythmias, meaning these are pertinent findings in CRS presentation, and a considerable level of heterogeneity (I^2^ > 90%) for fever and hypotension.

During the ICU stay, 60% CI-95% (11–99) of patients developed higher grades of CRS and 53% CI-95% (1–99) of ICANS. Notably, a considerable level of heterogeneity was reported among studies (I^2^ > 90%), probably due to the low number of studies assessing the development of CRS and ICANS in ICU and the spreading of the available data. (Figure 4).

**Figure 5:**
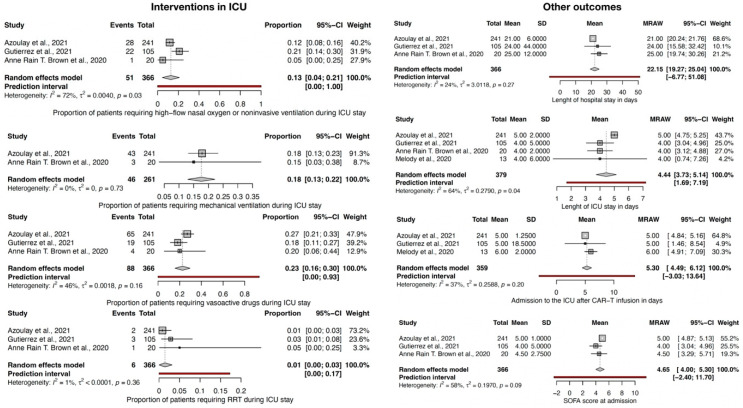
Forest plot for interventions in ICU and other outcomes [8,9,10,11].

As to the interventions initiated in ICU in CAR-T cell recipients (Figure 5), 13% CI-95% (4–21) required high-flow nasal oxygen or noninvasive ventilation, 18% CI-95% (13–22) required invasive mechanical ventilation, 23% CI-95% (16–30) required infusion of vasoactive drugs, and 1% CI-95% (0.1–3) required renal replacement therapy (RRT) during ICU stay. Mechanical ventilation, vasoactive drugs, and RRT had a low level of heterogeneity and *p* > 0.10 and could be considered as being salient features of this patient population. Only high-flow nasal oxygen or noninvasive ventilation had a substantial level of heterogeneity among the studies in this case.

The mean length of hospital stay was 22 days CI-95% (19–25), and admission to intensive care after CAR-T infusion was 5.3 days, 95%-CI (3.49; 6.12), with studies having a low level of heterogeneity with *p* > 0.10 (Figure 5).

SOFA score and length of ICU stay, in days, had a substantial level of heterogeneity (I^2^ around 60%), with a SOFA score, on average, of 4.65, 95%-CI (4–5.30) on admission and length of ICU of 4.5 days CI-95% (3.73–5.14).

Regarding publication bias, the results of funnel plots supported by Egger’s and Begg’s tests are available in Supplementary Files, but because of the few studies available, the power of the tests could be too low to distinguish chance from real asymmetry.

## 9. Discussion

We describe a meta-analysis of proportions on four studies comprising critically ill patients who received CAR-T therapy and required ICU admission. The results of this meta-analysis show that the quality of the available evidence is insufficient. There are no previous systematic reviews with meta-analyses focused on CAR-T cell recipients admitted to the ICU. Nonetheless, because many of the disease-related or treatment-related complications might lead to multiorgan failure, which could require intensive care admission, it is highly important to have an overview of the main outcomes in this population and to know what to expect, especially when dealing with this kind of patient for the first time. 

Regarding the mentioned studies, there was no information available about the overall admission policy of the ICU. 

Given the matter that around one-third of the total recipients of CAR-T cells required ICU admission, it represents an important fact to remember that highlights the need to have an available bed or dedicated high-dependency unit with trained medical personnel. Even though the incidence of ICU admission showed a substantial level of heterogeneity among the studies (I^2^ of 80%), this rate of admission was similar to the one mentioned in the literature [6,18,19]. Moreover, the readmission rate to the ICU within 30 days was around 18%, which suggests that physicians should expect readmission after an initial successful recovery in a significant proportion of patients. Reasons for readmission are not mentioned in the assessed studies, but they should be clearly stated for clinical and economic purposes.

Major reasons for ICU admission for patients treated with CAR-T cell are the development of CRS and ICANS, associated sepsis, single or multiple organ failure, hemophagocytic lymphohistiocytosis, stroke, and difficult airway management (bulky cervical or mediastinal lymphadenopathy, and tumor infiltration) [20,21]. CRS and ICANS were present in a third of these patients at the moment of admission to the ICU. Hypotension, altered mental status, AKI, and acute respiratory failure were the main reasons for ICU admission in these studies. This underlines that hematologists should carefully assess these patients daily on the wards for neurological, respiratory, cardiovascular, and kidney organ failure. Usually, the patients deteriorate clinically over time, and there is a failure to identify the process [22,23]. A delay in admission to the ICU and more than one organ failure are associated with increased mortality [24,25]. Validated scores such as MEWS/NEWS should be used daily by the medical personnel to assess the patients on the ward, and with the help of these, early admission to the ICU could be suggested [26,27,28]. 

As mentioned before, CRS is a complication of CAR-T cell treatment. Hypotension, AKI, arrhythmias, and fever are the most frequent signs and symptoms of CRS presentation. The presence of these signs and symptoms should prompt physicians to do a thorough screening for CRS, which could lead to organ failure if not taken care of. Noteworthy, the same signs and symptoms could be present in sepsis, so it is worth remembering that sepsis is a differential diagnosis when dealing with CRS. A comprehensive sepsis screening should rapidly follow with the institution of sepsis treatment if suspected [14,29]. The lymphodepletion regimen given prior to CAR-T cell therapy leads to immunosuppression, which can lead to bacterial infections. Patients with immunosuppression and CAR–T cell-associated toxicities have the highest risk for infection, which is reported to be around 23–43% during the first month after CAR-T therapy infusion [30,31]. Cytokine identification could be used to differentiate between CRS and sepsis. A study by Diorio et al. suggests a combination of IFNγ and IL1β, which could be able to categorize subjects as having CRS or sepsis with 97% accuracy, but this requires further research [32].

The most required interventions initiated in ICU in CAR-T cell recipients were high-flow nasal oxygen or noninvasive ventilation, invasive mechanical ventilation, infusion of vasoactive drugs, and a small proportion even required renal replacement therapy (RRT) during ICU stay. Notable, while AKI was one of the main reasons for ICU admission and one of the main signs of CRS presentation, RRT was only required in a low percentage of cases. The lack of diagnostic criteria for AKI within the assessed manuscripts makes it impossible to make assumptions. Future studies should focus on what is the real percentage of patients presenting with AKI and which one of the patients could be helped by initiating RRT early. RRT with Cytosorb^®^ or other hemadsorbers can be used as a bridge therapy for refractory CRS, as well as for managing sepsis, due to the elimination/adsorption of cytokines through the filter [33,34].

The mean length of hospital stay was around 22 days, ICU stay around 5 days, and admission to the intensive care after CAR-T infusion was around 5 days, so physicians should expect a relatively long duration of hospital stay, which increases costs. 

A mean SOFA value of 4.5 calculated at the admission moment corresponds to a mortality of around 15–20%, according to the literature [35,36]. Hematologic patients are considered to be a frail patient population [25,37]. Putting these two facts together, there is a suggestion that there is an increased risk of mortality among hematologic patients treated with CAR-T cells, even though the reported mortality of CAR-T cell recipients admitted to the ICU is around 6%, which could be considered low. A deeper look into this finding is required due to the fact that the mortality rate is disease, treatment, or sepsis-related, and the SOFA calculated in the mentioned studies is not solely performed on oncohematological patients. By having a dedicated team, environment, guidelines, and standard of care, the rate of detection of complications can increase, and earlier treatment started, which could improve the overall survival rate. 

Regarding the limitation of our study, first of all, the lack of overall prospective, randomized controlled studies with a high number of patients that evaluate the recipients of CAR-T cell therapies in ICU and the lack of databases from the available studies, impeded the creation of a more complex and complete database. Studies included in this meta-analysis were mainly retrospective, with a high degree of heterogeneity, and not all of them studied all the mentioned outcomes. Regarding publication bias, funnel plots supported by Egger’s and Begg’s tests were performed, but because of the few studies available, the power of the tests could be too low to distinguish chance from real asymmetry. Incomplete and scarce information regarding these patients treated in ICU contributes to the shortcomings and inability to create a joint standard of research and care. 

Due to the scarcity of the literature available and the heterogeneity of the sample, a final assured conclusion cannot be made, and more evidence has to be gathered. Thus far, the general information is presumptive. Therefore, we underline the importance of undertaking future studies in this field by following common variables.

The focus of ICU management of these patients should reside on several problems. For an optimal management strategy in cases of respiratory failure, a lung protective strategy and a review of chest and neck imaging in the hematologic population should become standard practice to avoid airway emergencies and adverse outcomes. Regarding fluid management, goal-directed fluid therapy should be followed and helped by the use of performing point-of-care cardiac and lung ultrasound to guide it [38]. The choice of the most appropriate intravenous fluid, like crystalloids or colloids, and the use of vasoactive agents remain to be studied in the setting of pulmonary capillary leak, CRS-related cardiomyopathy, or oliguric renal failure [39]. Unfortunately, when sepsis is present in these patients, there are arguments that it is correlated with the risk of particularly poor outcomes [9]. This suggests that a comprehensive search for the site of infections is mandatory.

Studies that focus on death from the progression of the disease in contrast to death from toxicity of treatment will have to be conducted to establish the incriminating factors. While immunotherapies are in continuous development, with newer CAR-T cell therapies entering the market, the indications for this treatment could become broader [1,40]. Toxicities and complications magnitude will have to be considered and anticipated because, at the moment, they are difficult to predict. 

Earlier admission to the ICU or high dependency unit (HDU) of patients at high risk of CRS might also improve outcomes, as seen in previous studies on hematological malignancies, and might paradoxically reduce the duration of stay in the ICU [41]. Having a hematological pathology does not seal access to an ICU, and it does not mean there are no benefits if hospitalized in the ICU, as many might think. The current risk stratification models in the context of immunotherapies are limited. There is not a single perfect tool for early prediction for ICU admission, but by adopting different scores like MEWS/NEWS and having a second opinion from an ICU physician, the threshold for admission could be set lower. Risk prediction models in the context of critically ill patients following immunotherapies could be developed by using clinical prediction models or machine learning algorithms.

There is a dire need to create a standard of care and research to change and improve the current practice, with approaches focusing on reducing the incidence of CRS and ICANS, the need for ICU admission, early sepsis screening, antimicrobial prophylaxis, and research on management strategies to enhance the care of critically ill patients following CAR-T cell therapy, characterization of the clinical course for future prevention planning, all of which work to improve the overall outcomes and increase the quality of life of these patients.

The current manuscript highlights that patients treated with CAR-T cell therapy have a high chance of ending up in the ICU. The main focus should be on approaching each case thoroughly, firstly as a dedicated ICU physician doing the rounds with a hematologist and secondly performing screening for CRS and sepsis at the moment of admission to the ICU. This study may help in organizing future studies, encourage the publishing of positive as well as negative results, and contribute to shaping evidence for the creation of a standard of care. 

Unfortunately, the assessed studies of this meta-analysis did not survey all of the outcomes available. Therefore, for those who want to assess the outcomes of CAR-T cell recipients after admission to the ICU, we propose they include the following variables in their studies. The reason is to find common ground and to make it easier for future meta-analyses. (Table 2).

- CAR T-cell therapy–associated TOXicity (CARTOX)
- ICU—Intensive care unit
- Modified Early Warning Score (MEWS)/National Early Warning Score (NEWS)
- CRS—Cytokine release syndrome
- ICANS—immune effector cell-associated neurotoxicity syndrome
- SOFA—Sequential Organ Failure Assessment score

Essentials:

Highlighting the baseline characteristics, main reasons for ICU admission, required ICU interventions, and ICU outcomes for patients who received CAR-T cell therapy and were admitted to the ICU. 

The first systematic review with meta-analysis of proportions focused on CAR-T cell recipients admitted to the ICU.

Addressing the importance of systematically conducted studies for a better understanding of the current practice and how to improve it in immunotherapies like CAR-T cell therapies. 

## 10. Conclusions

The endless opportunities that immunotherapy has to offer pose a challenge to both hematologists and intensive care physicians. The future is pictured as a worldwide standard of care as ICU management becomes integrated with overall treatment opportunities. More clinical trials are required to address the current state of things, with trials that should also address the different CAR-T therapies available. 

The findings of our study identify several outcomes in patients treated with CAR-T that need to be considered by intensive care physicians and highlight an urgent need for further research development and guidance for physicians, including other specialties, regarding high-quality interventions and the right time to admit a patient to the ICU.

## Figures and Tables

**Table 1:** Summary of study characteristics.

	First Author (Year)	Characteristic	Description	Conclusion
1	Azoulay et al. 2021 [9]	Duration Country Study design Outcome Type of CAR-T	1 February 2018–1 February 2020 International Multicenter, observational, retrospective, and prospective The primary endpoint was 90-day mortality Not mentioned 241 patients admitted to the ICU	A significant association between the admission diagnosis and 90-day mortality, which was 22.4% (95% CI 17.1–27.7), with increased mortality in patients who had presented with sepsis, frail patients, and when they required life-saving therapy within 24 h after admission to the ICU.
2	Gutierrez et al. 2021 [11]	Duration Country Study design Type of CAR-T	November 2017 and May 2019 U.S. Multicenter, retrospective, cohort Axicabtagene ciloleucel 105 patients were admitted to the ICU	The high cost of CAR-T and a higher rate of ICU admissions for this patient population and additional research to identify predictors of ICU and hospital mortality are needed to inform accurate prognostication in the CAR-T cell population.
3	Brown et al. 2020 [8]	Duration Country Study design Outcome Type of CAR-T	November 2017 and August 2018 The U.S. Single-center, retrospective Mortality up to 60 days Axicabtagene ciloleucel 20 patients admitted to the ICU	The high cost and higher rate of ICU admission for this patient population suggest this should not be a decision-guiding factor in limiting access to treatment due to the frequent reversible nature of complications and a higher rate of hospital discharge and survival.
4	Melody et al. 2020 [10]	Duration Country Study design Outcome Type of CAR-T	June 2018 and June 2020 U.S Retrospective Serum ferritin and CRP levels with length of ICU stay Axicabtagene ciloleucel 13 patients admitted to the ICU	C-reactive protein (CRP) and ferritin are serum inflammatory markers associated with the onset and persistence of CAR-T cell-related toxicity.

**Table 2:** Outcomes, predictors, and variables that should be included in future international, multicentre, prospective studies dealing with CAR-T cell recipients.

Baseline Characteristics	Outcomes
Authors Year Design of study Total patients Number of patients treated with CAR-T Age Sex Underlying malignancy Time since diagnosis of the malignancy, years Any comorbid condition Previous stem-cell transplantation (autologous/allogenic) Number of chemotherapy lines before CAR T-cell therapy Clinical diagnosis upon evaluation in the wards Frailty Type of CAR-T therapy Median time from infusion to peak Median follow-up of patients Neutropenia at ICU admission Median time from CAR T-cell infusion to death	Length of hospital stay, days Length of ICU stay, days Hospital to ICU admission, days Longer follow-up 90-day mortality across all participants 60-day mortality 30-day mortality Hospital mortality Intensive care unit mortality Number of ICU-admitted patients Readmission to the ICU within 30 days Death due to sepsis Disease-related death Treatment-related death Admission to intensive care after CAR-T infusion
SCORES	CAR-T toxicities—CRS and ICANS
CARTOX SCORE Frailty scores MEWS/NEWS on the ward	CRS grade CRS symptoms CRS during ICU stay Incidence of isolated CRS ICANS grade ICANS symptoms ICANS grade in the ICU ICANS during their ICU stay Incidence of isolated ICANS Incidence of CRS and ICANS
Intensive care unit	SEPSIS
SOFA Reasons for ICU admission (hypotension, sepsis, acute kidney injury, acute respiratory failure, coma, arrhythmias, etc.) Need for life-saving therapy at ICU admission—mechanical ventilation, vasoactive drugs, renal replacement therapy Treatment used in ICU Strategies to improve timely admission to the ICU Prognosis factors Duration of life-supporting interventions	Antibiotics used/sepsis screening microbiologically documented infection clinically suspected infection C-reactive protein concentration, mg/L Ferritin concentration, μg/L Other markers (procalcitonin, presepsin, etc.) Infections before and during ICU Citokine measurement (IL-1, IL-6, etc.

## Data Availability

All data are available, either analyzed as figures and tables presented in the current manuscript or as raw data upon request by any external collaborator or reviewer.

## Supplementary Materials

The following supporting information can be downloaded at https://www.mdpi.com/article/10.3390/jcm12186098/s1.
