# Supplementary material for: Outcomes of CAR-T Cell Therapy Recipients Admitted to the ICU: In Search for a Standard of Care—A Brief Overview and Meta-Analysis of Proportions
Source: J Clin Med. 2023 Sep 21;12(18):6098. doi: 10.3390/jcm12186098 (PMC10531736; doi:10.3390/jcm12186098)
Supplement: Supplementary file 1 [file jcm-12-06098-s001.zip › jcm-2594642-supplementary.pdf]

Supplemental document

Table of Contents

Statistics ***3***

1. ICU Admission – Male sex ***3***

Test for heterogeneity 4

Publication bias 4

2. ICU admitted ***4***

Test for heterogeneity 5

Publication bias 5

3. ICU mortality ***6***

Test for heterogeneity 6

Publication bias 6

4. Hospital mortality ***6***

Test for heterogeneity 7

Publication bias 7

5. Reasons for ICU admission – hypotension ***7***

Test for heterogeneity 8

Publication bias 8

6. Reasons for ICU admission – acute kidney injury ***8***

Test for heterogeneity 9

Publication bias 9

7. Reasons ICU admission – acute respiratory failure ***9***

Test for heterogeneity 10

Publication bias 10

8. Reasons for ICU admission – altered mental status ***10***

Test for heterogeneity 11

Publication bias 11

9. Number of chemotherapy lines before CAR-T infusion ***11***

10. Isolated CRS ***12***

Test for heterogeneity 12

Publication bias 13

11. Isolated ICANS 13
Test for heterogeneity 13
Publication bias 13
12. CRS and ICANS 14
Test for heterogeneity 14
Publication bias 14
13. CRS presentation - fever 15
Test for heterogeneity 15
Publication bias 15
14. CRS presentation - hypotension 15
Test for heterogeneity 16
Publication bias 16
15. CRS presentation – arrhythmias 16
Test for heterogeneity 17
Publication bias 17
16. CRS presentation – acute kidney injury 17
Test for heterogeneity 18
Publication bias 18
17. CRS developed during ICU stay 18
Test for heterogeneity 19
Publication bias 19
18. ICANS developed during ICU stay 20
Test for heterogeneity 20
Publication bias 20
19. High-flow nasal oxygen, non-invasive ventilation required 21
Test for heterogeneity 21
Publication bias 21
20. Vasoactive drugs 21
Test for heterogeneity 22
Publication bias 22
21. Renal replacement therapy 22
Test for heterogeneity 23
Publication bias 23
22. Mechanical ventilation during ICU stay 23

Test for heterogeneity 24
Publication bias 24
23. Readmission to the ICU within 30 days 25
Test for heterogeneity 25
Publication bias 25
24. Malignancy 26
Test for heterogeneity 26
Publication bias 26

Statistics

1. ICU Admission – Male sex

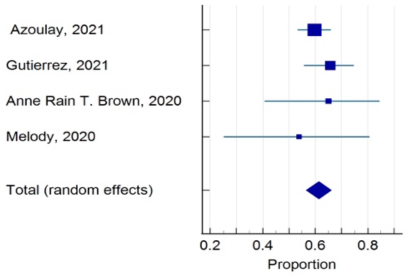

Study	Sample size	Proportion (%)	95% CI	Weight (%)	
					Random
Azoulay, 2021	241	59.751	53.264 to 65.996		63.19
Gutierrez, 2021	105	65.714	55.814 to 74.703		27.68
Anne Rain T. Brown, 2020	20	65.000	40.781 to 84.609		5.48
Melody, 2020	13	53.846	25.135 to 80.777		3.66
Total (random effects)	379	61.382	56.457 to 66.193	100.00	100.00

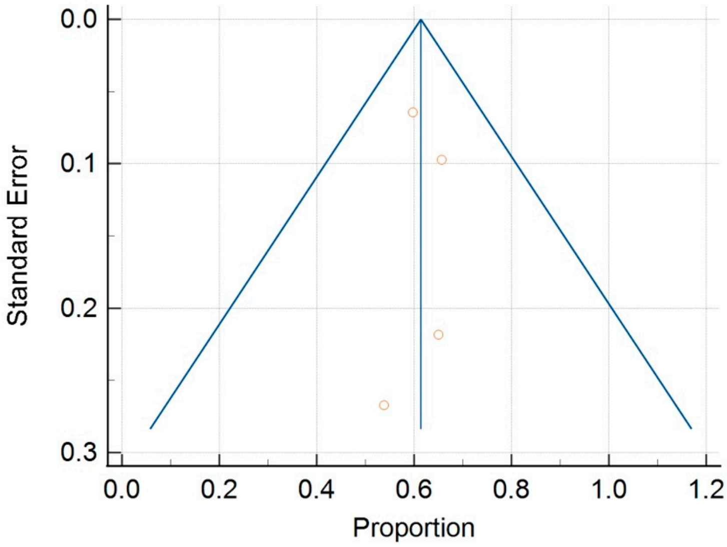

Test for heterogeneity

Q	1.5105
DF	3
Significance level	P = 0.6799
I^2 (inconsistency)	0.00%
95% CI for I^2	0.00 to 74.36

Publication bias

Egger's test	
Intercept	0.05185
95% CI	-3.7805 to 3.8842
Significance level	P = 0.9589
**Begg's test**	
Kendall's Tau	0.0000
Significance level	P = 1.0000

Figure 1. Forest plot of proportions of male patients admitted in the ICU, in published studies.

Figure 1 shows the combined proportion for our outcome of interest – the males admitted to the ICU as a percentage. On average, 61% CI 95%[56-66] of the recipients of CAR-T cells who were admitted to the ICU were males.

2. ICU admitted

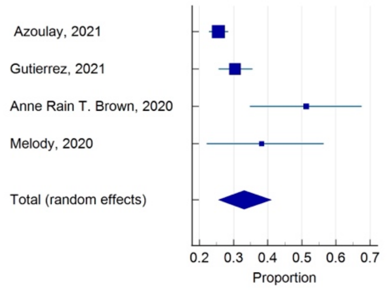

Study	Sample size	Proportion (%)	95% CI	Weight (%)
				Random
Azoulay, 2021	942	25.584	22.824 to 28.497	35.82
Gutierrez, 2021	345	30.435	25.621 to 35.588	32.81
Anne Rain T. Brown, 2020	39	51.282	34.780 to 67.582	16.30
Melody, 2020	34	38.235	22.167 to 56.436	15.07
Total (random effects)	1360	33.103	25.566 to 41.103	100.00

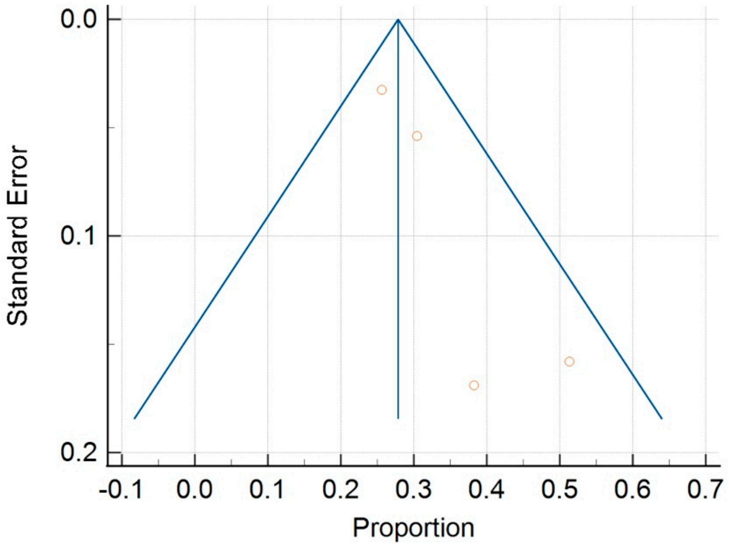

Test for heterogeneity

Q	14.7777
DF	3
Significance level	P = 0.0020
I^2 (inconsistency)	79.70%
95% CI for I^2	46.10 to 92.35

Publication bias

Egger's test	
Intercept	3.2312
95% CI	-0.6730 to 7.1354
Significance level	P = 0.0706
Begg's test	
Kendall's Tau	0.6667
Significance level	P = 0.1742

Figure 2. Forest plot of proportions of ICU admitted patients, in published studies.

Figure 2 shows the combined proportion for our outcome of interest – patients admitted to the ICU from total CAR-T recipients, as a percentage. On average, 33% CI-95% [26-41] of the recipients of CAR-T cells were admitted to the ICU. A substantial level of heterogeneity (80%) was reported.

3. ICU mortality

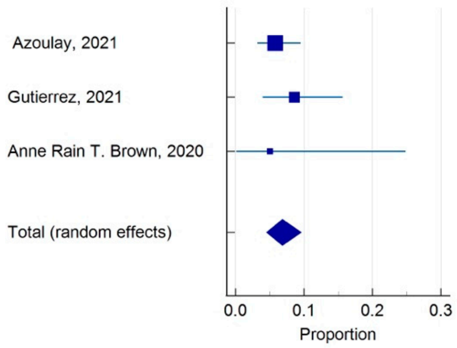

Study	Sample size	Proportion (%)	95% CI	Weight (%)
				Random
Azoulay, 2021	241	5.809	3.212 to 9.555	65.58
Gutierrez, 2021	105	8.571	3.995 to 15.648	28.73
Anne Rain T. Brown, 2020	20	5.000	0.127 to 24.873	5.69
Total (random effects)	366	6.842	4.495 to 9.638	100.00

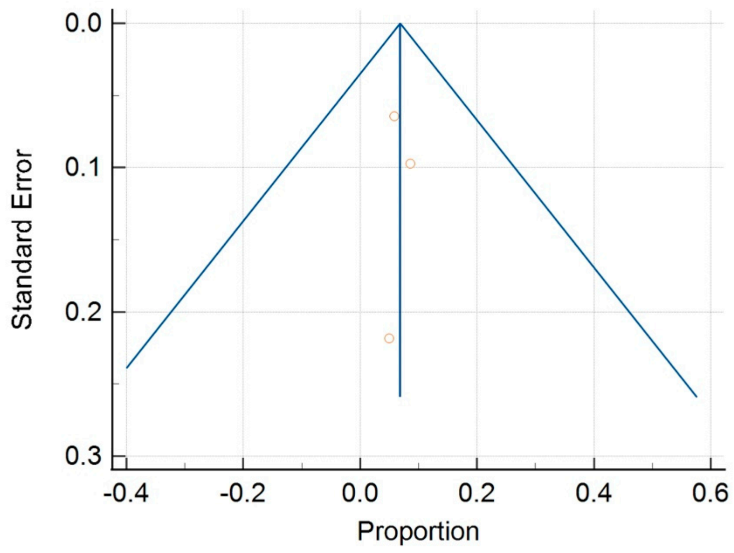

Test for heterogeneity

Q	0.9476
DF	2
Significance level	P = 0.6226
I^2 (inconsistency)	0.00%
95% CI for I^2	0.00 to 92.92

Publication bias

Egger's test	
Intercept	0.5875
95% CI	-15.4314 to 16.6065
Significance level	P = 0.7224
Begg's test	
Kendall's Tau	0.3333
Significance level	P = 0.6015

Figure 3. Forest plot of proportions of ICU admitted patients who died, in published studies.

Figure 3 shows the combined proportion for our outcome of interest – patients admitted to the ICU who died from total CAR-T recipients admitted in the ICU, as a percentage. On average, 7% CI-95% [5-10] of the recipients of CAR-T therapy who were admitted to the ICU died. A 0% level of heterogeneity was reported with a p>0.10.

4. Hospital mortality

Study	Sample size	Proportion (%)	95% CI	Weight (%)

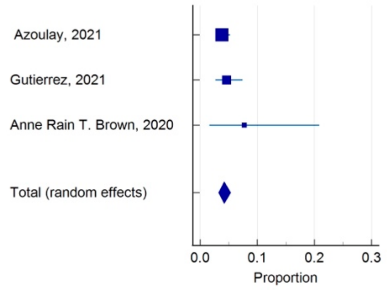

				Random
Azoulay, 2021	942	3.822	2.691 to 5.252	70.96
Gutierrez, 2021	345	4.638	2.674 to 7.422	26.03
Anne Rain T. Brown, 2020	39	7.692	1.615 to 20.870	3.01
Total (random effects)	1326	4.217	3.204 to 5.364	100.00

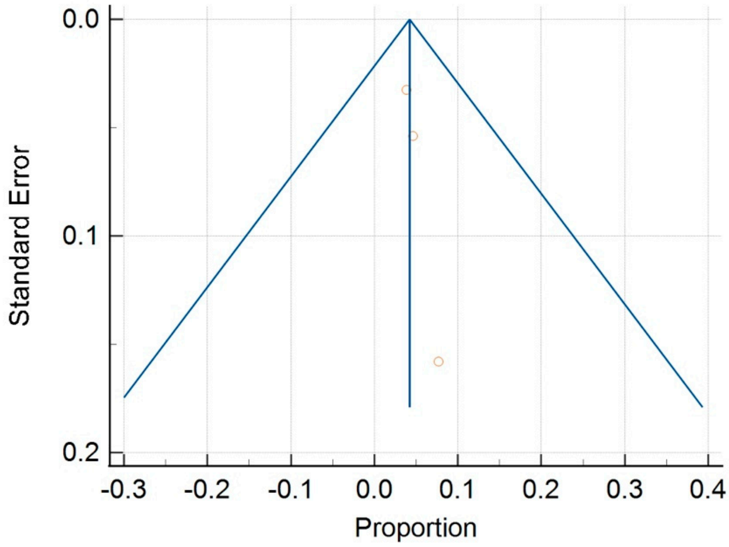

Test for heterogeneity

Q	1.9133
DF	2
Significance level	P = 0.3842
I² (inconsistency)	0.00%
95% CI for I²	0.00 to 96.49

Publication bias

Egger's test	
Intercept	1.6785
95% CI	-0.6556 to 4.0127
Significance level	P = 0.0694
Begg's test	
Kendall's Tau	1.0000
Significance level	P = 0.1172

Figure 4. Forest plot of proportions of CAR-T recipients who died in the hospital, in published studies.

Figure 4 shows the combined proportion for our outcome of interest – CAR-T recipients patients who died in the hospital, as a percentage. On average, 4% CI-95% [3-5] of the CAR-T recipients died in the hospital. A 0% level of heterogeneity was reported with a p>0.10.

5. Reasons for ICU admission – hypotension

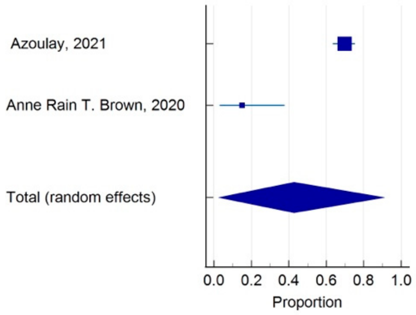

Study	Sample size	Proportion (%)	95% CI	Weight (%)
				Random
Azoulay, 2021	241	69.710	63.483 to 75.444	51.69
Anne Rain T. Brown, 2020	20	15.000	3.207 to 37.893	48.31
Total (random effects)	261	42.780	2.448 to 91.152	100.00

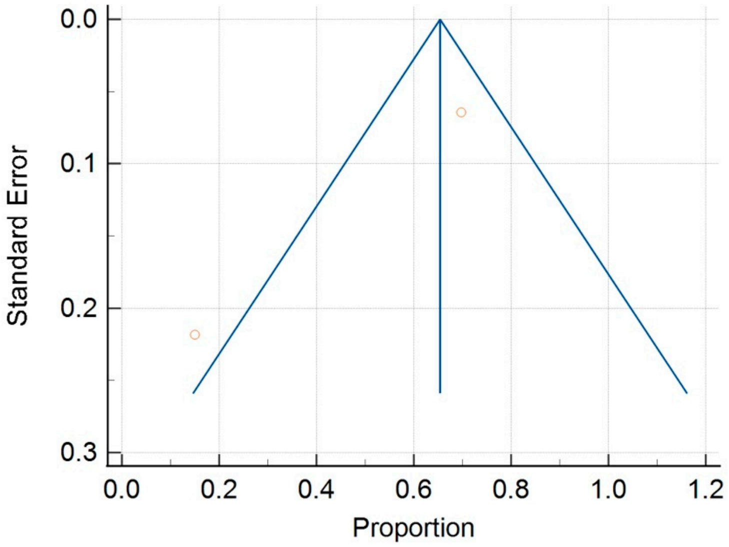

Test for heterogeneity

Q	24.8920
DF	1
Significance level	P < 0.0001
I^2 (inconsistency)	95.98%
95% CI for I^2	88.63 to 98.58

Publication bias

Egger's test	
Intercept	-7.3731
95% CI	
Significance level	P < 0.0001
Begg's test	
Kendall's Tau	-1.0000
Significance level	P = 0.3173

Figure 5. Forest plot of proportions of CAR-T recipients in which the main reason for admission to the ICU was hypotension, in published studies.

Figure 5 shows the combined proportion for our outcome of interest – hypotension as the main reason for admission to the ICU, as a percentage. On average, 42% CI-95% [3-91] of the CAR-T recipients were admitted to the ICU because of hypotension. A considerable level of heterogeneity was reported (96%).

6. Reasons for ICU admission – acute kidney injury

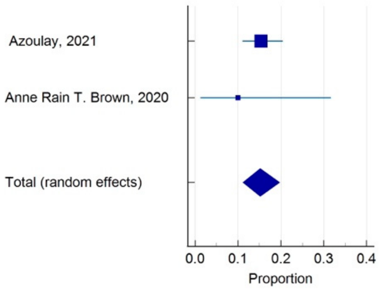

Study	Sample size	Proportion (%)	95% CI	Weight (%)
				Random
Azoulay, 2021	241	15.353	11.046 to 20.535	92.02
Anne Rain T. Brown, 2020	20	10.000	1.235 to 31.698	7.98
Total (random effects)	261	15.185	11.112 to 19.766	100.00

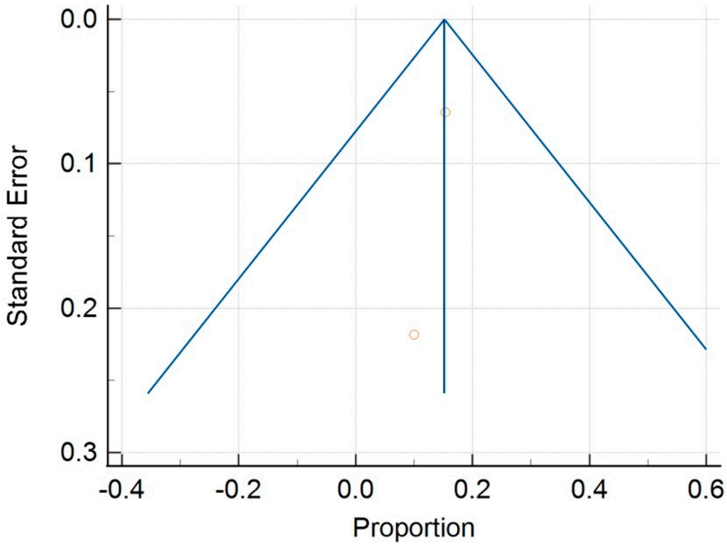

Test for heterogeneity

Q	0.2248
DF	1
Significance level	P = 0.6354
I^2 (inconsistency)	0.00%
95% CI for I^2	0.00 to 0.00

Publication bias

Egger's test	
Intercept	-0.7006
95% CI	
Significance level	P < 0.0001
Begg's test	
Kendall's Tau	-1.0000
Significance level	P = 0.3173

Figure 6. Forest plot of proportions of CAR-T recipients in which the main reason for admission to the ICU was acute kidney injury (AKI), in published studies.

Figure 6 shows the combined proportion for our outcome of interest – AKI as the main reason for admission to the ICU, as a percentage. On average, 15% CI-95% [11-19] of the CAR-T recipients were admitted to the ICU because of AKI. A 0% level of heterogeneity was reported with a p>0.10.

7. Reasons ICU admission – acute respiratory failure

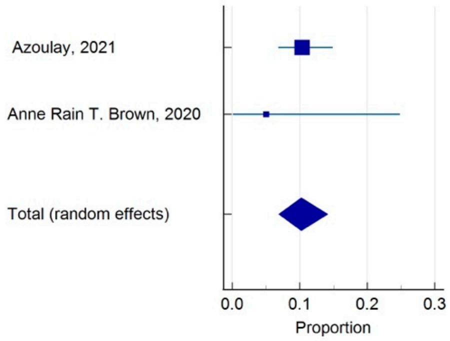

Study	Sample size	Proportion (%)	95% CI	Weight (%)
				Random
Azoulay, 2021	241	10.373	6.826 to 14.932	92.02
Anne Rain T. Brown, 2020	20	5.000	0.127 to 24.873	7.98
Total (random effects)	261	10.226	6.863 to 14.169	100.00

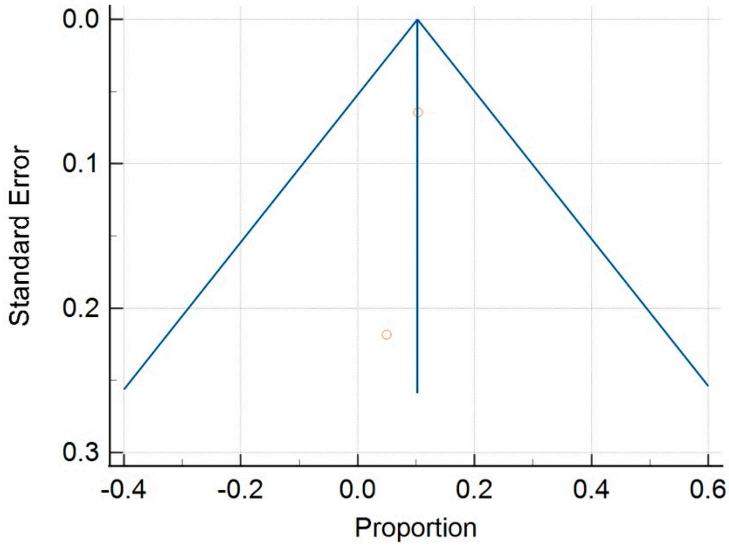

Test for heterogeneity

Q	0.3139
DF	1
Significance level	P = 0.5753
I^2 (inconsistency)	0.00%
95% CI for I^2	0.00 to 0.00

Publication bias

Egger's test	
Intercept	-0.8280
95% CI	
Significance level	P < 0.0001
Begg's test	
Kendall's Tau	-1.0000
Significance level	P = 0.3173

Figure 7. Forest plot of proportions of CAR-T recipients in which the main reason for admission to the ICU was acute respiratory failure, in published studies.

Figure 7 shows the combined proportion for our outcome of interest – acute respiratory failure as the main reason for admission to the ICU, as a percentage. On average, 10% CI-95% [7-14] of the CAR-T recipients were admitted to the ICU because of acute respiratory failure. A 0% level of heterogeneity was reported with a p>0.10.

8. Reasons for ICU admission – altered mental status

Study	Sample size	Proportion (%)	95% CI	Weight (%)

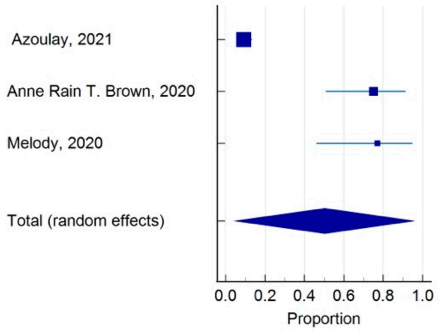

				Random
Azoulay, 2021	241	9.129	5.810 to 13.495	34.54
Anne Rain T. Brown, 2020	20	75.000	50.895 to 91.343	33.10
Melody, 2020	13	76.923	46.187 to 94.962	32.36
Total (random effects)	274	50.308	4.425 to 95.825	100.00

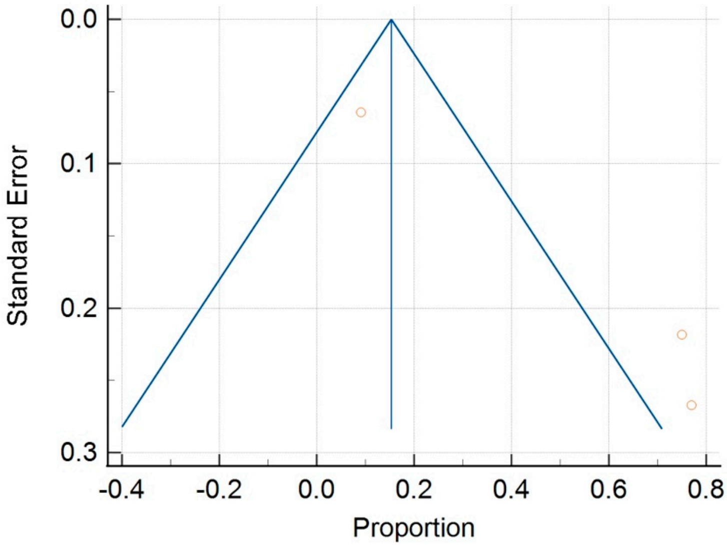

Test for heterogeneity

Q	65.1527
DF	2
Significance level	P < 0.0001
I^2 (inconsistency)	96.93%
95% CI for I^2	93.79 to 98.48

Publication bias

Egger's test	
Intercept	8.2422
95% CI	-6.1767 to 22.6611
Significance level	P = 0.0871
Begg's test	
Kendall's Tau	0.3333
Significance level	P = 0.6015

Figure 8. Forest plot of proportions of CAR-T recipients in which the main reason for admission to the ICU was altered mental status, in published studies.

Figure 8 shows the combined proportion for our outcome of interest – altered mental status as the main reason for admission to the ICU, as a percentage. On average, 50% CI-95% [4-95] of the CAR-T recipients were admitted to the ICU because of altered mental status. A considerable level of heterogeneity was reported (96%).

9. Number of chemotherapy lines before CAR-T infusion

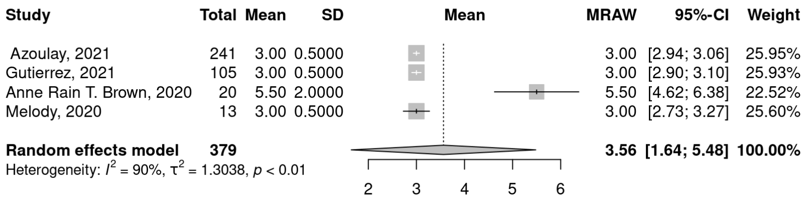

Figure 9. Forest plot of proportions of the number of chemotherapy lines patients received before CAR-T infusion, in published studies.

Figure 9 shows the combined proportion for our outcome of interest – the number of chemotherapy lines patients received before CAR-T infusion, as a crude mean. On average, the number of chemotherapy lines patients received before CAR-T infusion was 3,56, CI-95% [1,64; 5,48]. A considerable level of heterogeneity was reported (90%).

10. Isolated CRS

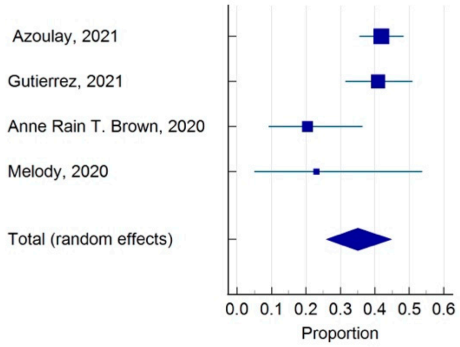

Study	Sample size	Proportion (%)	95% CI	Weight (%)
				Random
Azoulay, 2021	241	41.909	35.606 to 48.413	36.79
Gutierrez, 2021	105	40.952	31.451 to 50.978	31.02
Anne Rain T. Brown, 2020	39	20.513	9.296 to 36.464	21.24
Melody, 2020	13	23.077	5.038 to 53.813	10.95
Total (random effects)	398	35.071	25.854 to 44.887	100.00

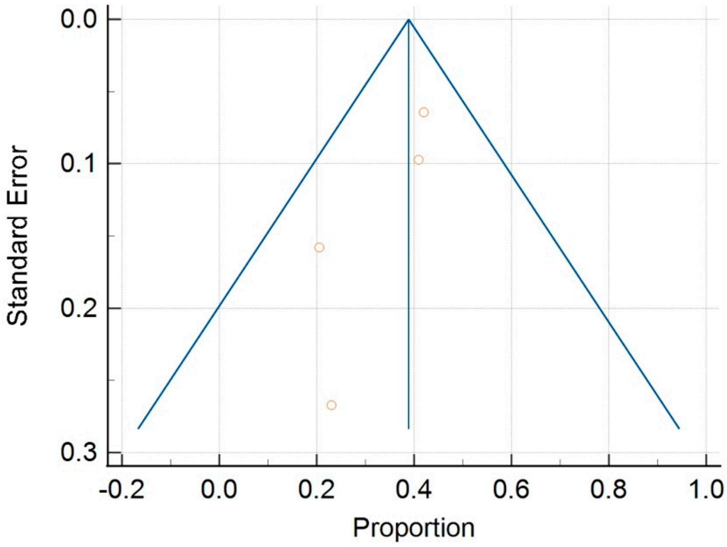

Test for heterogeneity

Q	8.4614

DF	3
Significance level	P = 0.0374

I² (inconsistency)	64.55%
95% CI for I²	0.00 to 87.99

Publication bias

Egger's test	
Intercept	-2.7338
95% CI	-8.1989 to 2.7313
Significance level	P = 0.1643
Begg's test	
Kendall's Tau	-0.6667
Significance level	P = 0.1742

Figure 10. Forest plot of proportions of CAR-T recipients who were admitted to the ICU with isolated CRS, in published studies.

Figure 10 shows the combined proportion for our outcome of interest – isolated CRS at admission to the ICU, as a percentage. On average, 35% CI-95% [26-45] of the CAR-T recipients were admitted to the ICU with isolated CRS. A substantial level of heterogeneity was reported (65%).

11.Isolated ICANS

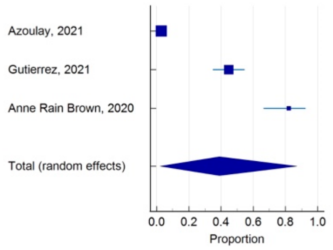

Study	Sample size	Proportion (%)	95% CI	Weight (%)
				Random
241	241	2.905	1.176 to 5.893	33.67
105	105	44.762	35.047 to 54.780	33.46
39	39	82.051	66.465 to 92.465	32.87
Total (random effects)	385	38.970	2.084 to 87.105	100.00

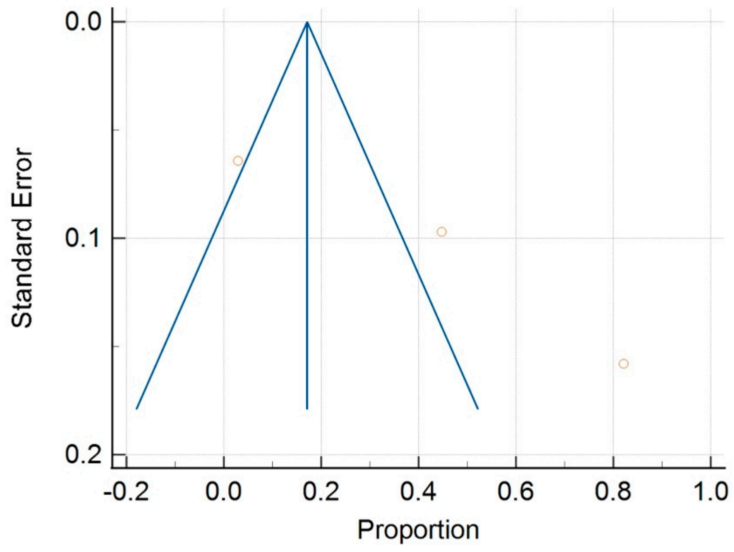

Test for heterogeneity

Q	177.9161
DF	2
Significance level	P < 0.0001
I² (inconsistency)	98.88%
95% CI for I²	98.12 to 99.33

Publication bias

Egger's test	
Intercept	22.2152
95% CI	-61.0877 to 105.5181
Significance level	P = 0.1827
Begg's test	
Kendall's Tau	1.0000
Significance level	P = 0.1172

Figure 11. Forest plot of proportions of CAR-T recipients who were admitted to the ICU with isolated ICANS, in published studies.

Figure 11 shows the combined proportion for our outcome of interest – isolated ICANS at admission to the ICU, as a percentage. On average, 39% CI-95% [2-87] of the CAR-T recipients were admitted to the ICU with isolated ICANS. A considerable level of heterogeneity was reported (99%).

12. CRS and ICANS

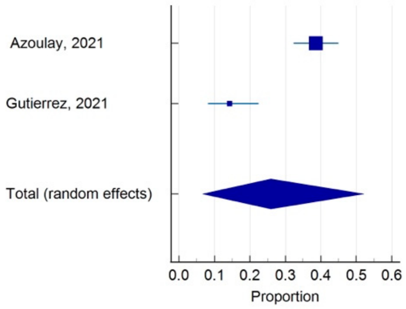

Study	Sample size	Proportion (%)	95% CI	Weight (%)
				Random
Azoulay, 2021	241	38.589	32.410 to 45.053	50.85
Gutierrez, 2021	105	14.286	8.222 to 22.465	49.15
Total (random effects)	346	25.900	6.665 to 52.133	100.00

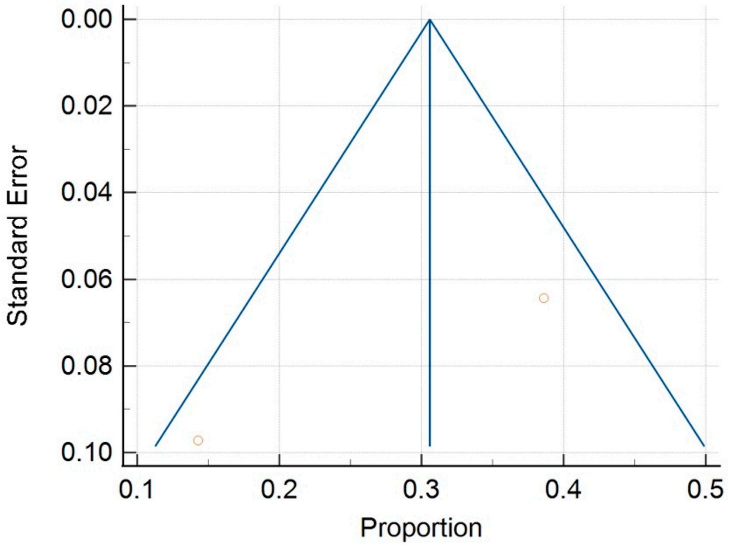

Test for heterogeneity

Q	22.8555
DF	1
Significance level	P < 0.0001
I^2 (inconsistency)	95.62%
95% CI for I^2	87.31 to 98.49

Publication bias

Egger's test	
Intercept	-16.9527
95% CI	
Significance level	P < 0.0001
Begg's test	
Kendall's Tau	-1.0000
Significance level	P = 0.3173

Figure 12. Forest plot of proportions of CAR-T recipients who were admitted to the ICU with CRS and ICANS, in published studies.

Figure 12 shows the combined proportion for our outcome of interest – CRS and ICANS at admission to the ICU, as a percentage. On average, 26% CI-95% [7-52] of the CAR-T recipients were admitted to the ICU with CRS and ICANS. A considerable level of heterogeneity was reported (95%).

13.CRS presentation - fever

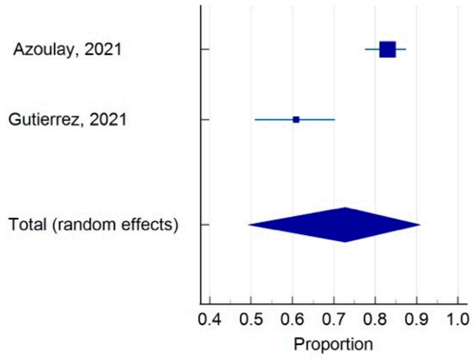

Study	Sample size	Proportion (%)	95% CI	Weight (%)
				Random
Azoulay, 2021	241	82.988	77.637 to 87.506	51.07
Gutierrez, 2021	105	60.952	50.944 to 70.326	48.93
Total (random effects)	346	72.784	49.253 to 90.994	100.00

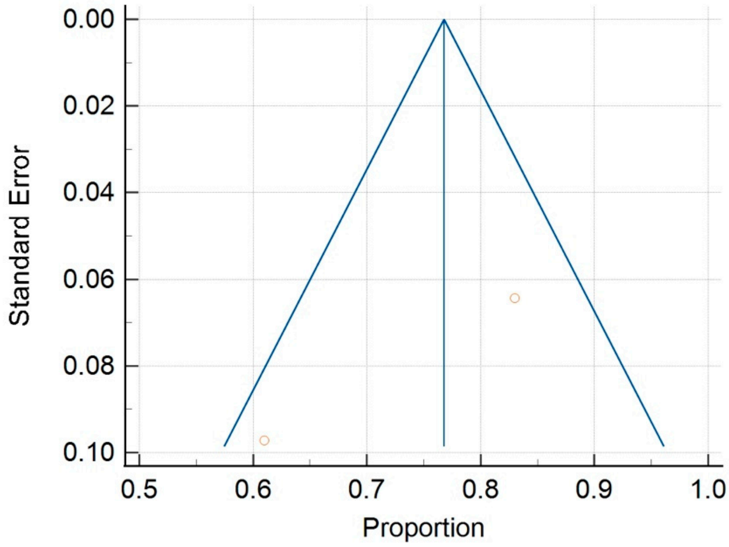

Test for heterogeneity

Q	18.2918
DF	1
Significance level	P < 0.0001
I^2 (inconsistency)	94.53%
95% CI for I^2	83.10 to 98.23

Publication bias

Egger's test	
Intercept	-15.1661
95% CI	
Significance level	P < 0.0001
Begg's test	
Kendall's Tau	-1.0000
Significance level	P = 0.3173

Figure 13. Forest plot of proportions of CAR-T recipients who were admitted to the ICU with fever as the main symptom of CRS, in published studies.

Figure 13 shows the combined proportion for our outcome of interest – fever as the main symptom of CRS at admission to the ICU, as a percentage. On average, 72% CI-95% [49-91] of the CAR-T recipients were admitted to the ICU with fever as the main symptom of CRS. A considerable level of heterogeneity was reported (95%).

14. CRS presentation - hypotension

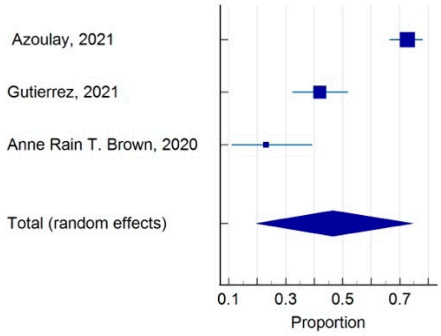

Study	Sample size	Proportion (%)	95% CI	Weight (%) Random
Azoulay, 2021	241	72.614	66.521 to 78.142	34.42
Gutierrez, 2021	105	41.905	32.345 to 51.933	33.73
Anne Rain T. Brown, 2020	39	23.077	11.134 to 39.326	31.85
Total (random effects)	385	46.523	19.602 to 74.596	100.00

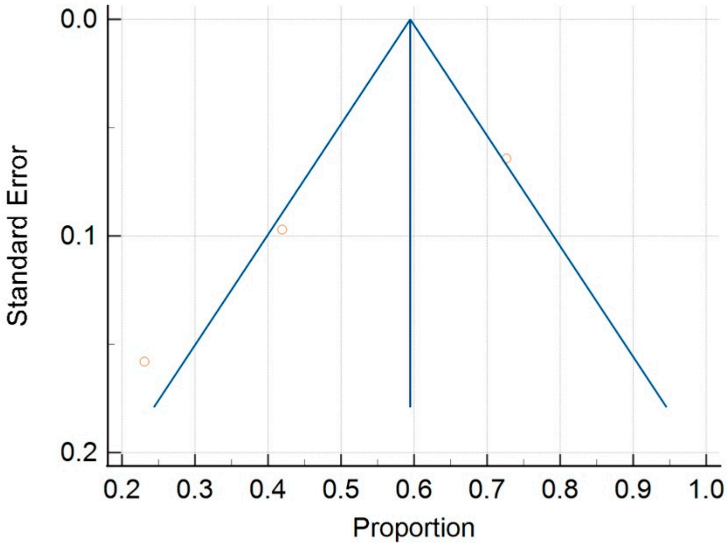

Test for heterogeneity

Q	53.7458
DF	2
Significance level	P < 0.0001
I^2 (inconsistency)	96.28%
95% CI for I^2	92.15 to 98.24

Publication bias

Egger's test	
Intercept	-12.1037
95% CI	-62.2401 to 38.0328
Significance level	P = 0.2006
Begg's test	
Kendall's Tau	-1.0000
Significance level	P = 0.1172

Figure 14. Forest plot of proportions of CAR-T recipients who were admitted to the ICU with hypotension as the main symptom of CRS, in published studies.

Figure 14 shows the combined proportion for our outcome of interest – hypotension as the main symptom of CRS at admission to the ICU, as a percentage. On average, 46% CI-95% [20-75] of the CAR-T recipients were admitted to the ICU with hypotension as the main symptom of CRS. A considerable level of heterogeneity was reported (96%).

15.CRS presentation – arrhythmias

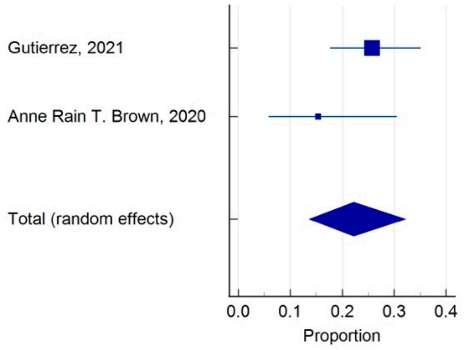

Study	Sample size	Proportion (%)	95% CI	Weight (%)
				Random
Gutierrez, 2021	105	25.714	17.680 to 35.165	63.57
Anne Rain T. Brown, 2020	39	15.385	5.862 to 30.528	36.43
Total (random effects)	144	22.214	13.608 to 32.231	100.00

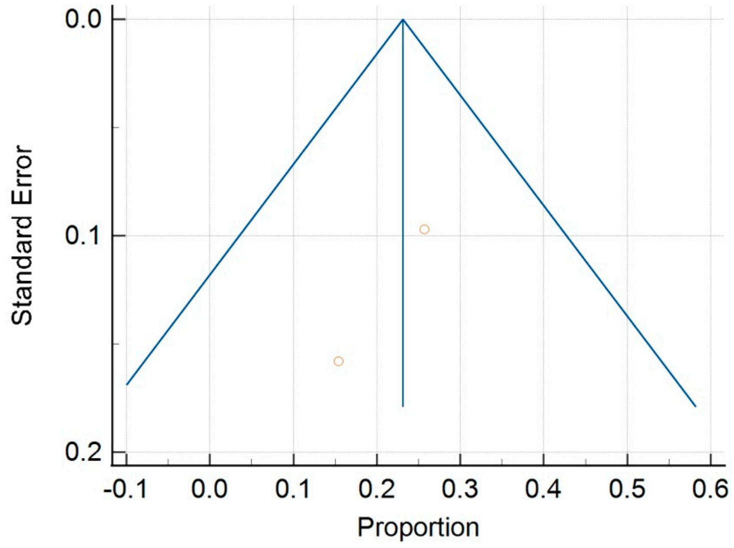

Test for heterogeneity

Q	1.6660
DF	1
Significance level	P = 0.1968
I^2 (inconsistency)	39.98%
95% CI for I^2	0.00 to 0.00

Publication bias

Egger's test	
Intercept	-3.9274
95% CI	
Significance level	P < 0.0001
Begg's test	
Kendall's Tau	-1.0000
Significance level	P = 0.3173

Figure 15. Forest plot of proportions of CAR-T recipients who were admitted to the ICU with arrhythmias as the main symptom of CRS, in published studies.

Figure 15 shows the combined proportion for our outcome of interest – arrhythmias as the main symptom of CRS at admission to the ICU, as a percentage. On average, 22% CI-95% [14-32] of the CAR-T recipients were admitted to the ICU with arrhythmias as the main symptom of CRS. A moderate level of heterogeneity was reported (40%) with a p>0.10.

16.CRS presentation – acute kidney injury

Study	Sample size	Proportion (%)	95% CI	Weight (%)

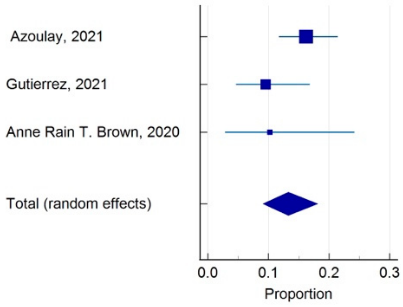

				Random
Azoulay, 2021	241	16.183	11.768 to 21.451	51.76
Gutierrez, 2021	105	9.524	4.662 to 16.818	32.61
Anne Rain T. Brown, 2020	39	10.256	2.866 to 24.221	15.63
Total (random effects)	385	13.282	9.078 to 18.144	100.00

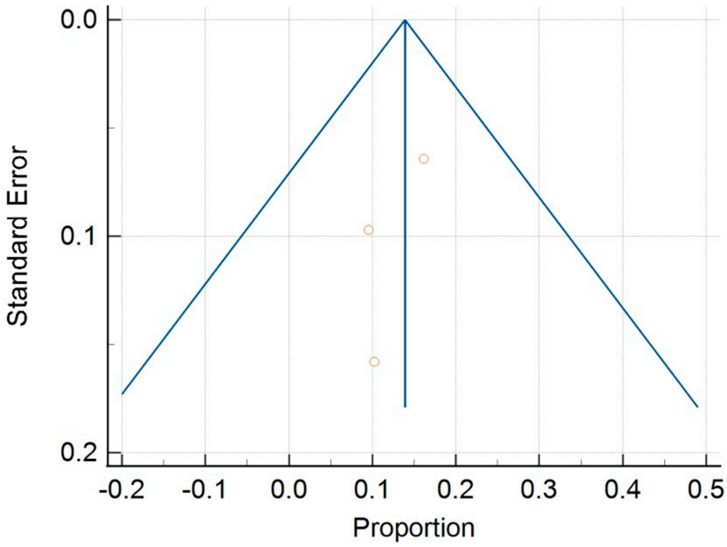

Test for heterogeneity

Q	2.9984
DF	2
Significance level	P = 0.2233
I^2 (inconsistency)	33.30%
95% CI for I^2	0.00 to 97.76

Publication bias

Egger's test	
Intercept	-2.2177
95% CI	-28.0187 to 23.5833
Significance level	P = 0.4720
Begg's test	
Kendall's Tau	-0.3333
Significance level	P = 0.6015

Figure 16. Forest plot of proportions of CAR-T recipients who were admitted to the ICU with AKI as the main symptom of CRS, in published studies.

Figure 16 shows the combined proportion for our outcome of interest – AKI as the main symptom of CRS at admission to the ICU, as a percentage. On average, 13% CI-95% [9-18] of the CAR-T recipients were admitted to the ICU with AKI as the main symptom of CRS. A low level of heterogeneity was reported (33%) with a p>0.10.

17.CRS developed during ICU stay

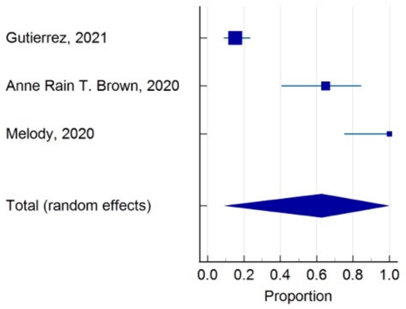

Study	Sample size	Proportion (%)	95% CI	Weight (%)
				Random
Gutierrez, 2021	105	15.238	8.968 to 23.563	34.32
Anne Rain T. Brown, 2020	20	65.000	40.781 to 84.609	33.18
Melody, 2020	13	100.000	75.295 to 100.000	32.50
Total (random effects)	138	62.760	9.366 to 99.720	100.00

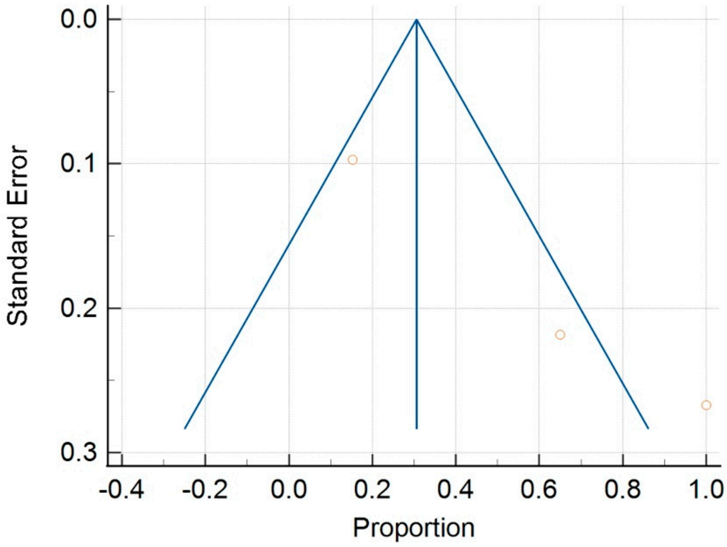

Test for heterogeneity

Q	64.1914
DF	2
Significance level	P < 0.0001
I^2 (inconsistency)	96.88%
95% CI for I^2	93.67 to 98.47

Publication bias

Egger's test	
Intercept	10.7162
95% CI	-14.0078 to 35.4402
Significance level	P = 0.1144
Begg's test	
Kendall's Tau	1.0000
Significance level	P = 0.1172

Figure 17. Forest plot of proportions of CAR-T recipients who developed CRS during ICU stay, in published studies.

Figure 17 shows the combined proportion for our outcome of interest – patients who developed CRS during ICU stay, as a percentage. On average, 62% CI-95% [9,3-99] of the CAR-T recipients developed CRS during ICU stay. A considerable level of heterogeneity was reported (97%).

18.ICANS developed during ICU stay

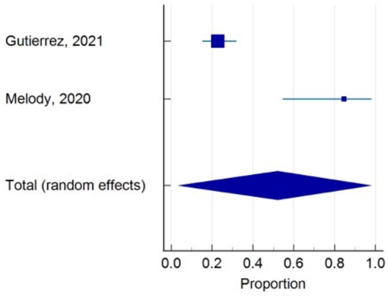

Study	Sample size	Proportion (%)	95% CI	Weight (%)
				Random
Gutierrez, 2021	105	22.857	15.227 to 32.074	51.92
Melody, 2020	13	84.615	54.553 to 98.079	48.08
Total (random effects)	118	52.132	3.385 to 97.987	100.00

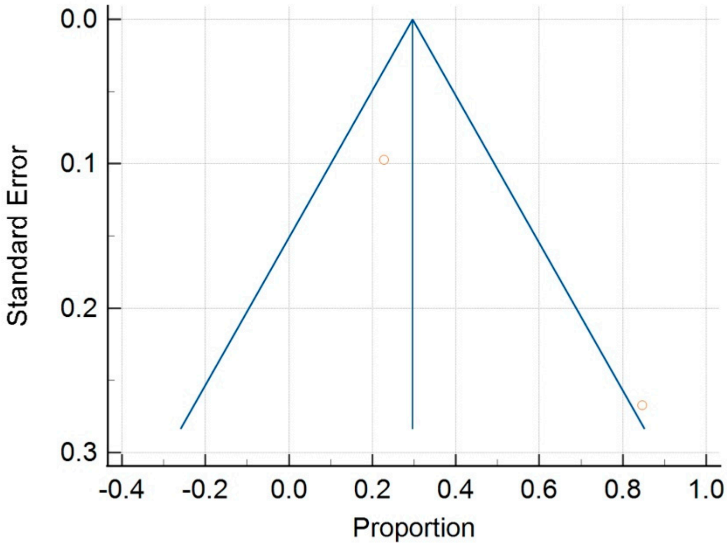

Test for heterogeneity

Q	19.9372
DF	1
Significance level	P < 0.0001
I^2 (inconsistency)	94.98%
95% CI for I^2	84.88 to 98.34

Publication bias

Egger's test	
Intercept	7.4631
95% CI	
Significance level	P < 0.0001
Begg's test	
Kendall's Tau	1.0000
Significance level	P = 0.3173

Figure 18. Forest plot of proportions of CAR-T recipients who developed ICANS during ICU stay, in published studies.

Figure 18 shows the combined proportion for our outcome of interest – patients who developed ICANS during ICU stay, as a percentage. On average, 52% CI-95% [3,3-98] of the CAR-T recipients developed ICANS during their ICU stay. A considerable level of heterogeneity was reported (95%).

19. High-flow nasal oxygen, non-invasive ventilation required

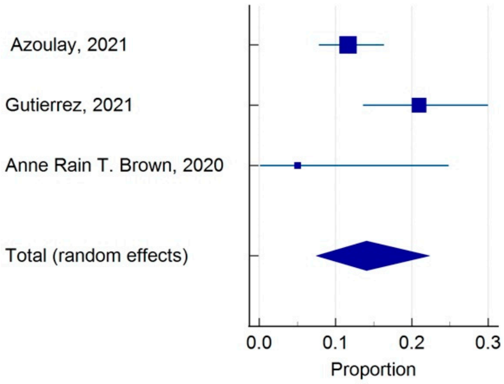

Study	Sample size	Proportion (%)	95% CI	Weight (%)
				Random
Azoulay, 2021	241	11.618	7.861 to 16.352	45.11
Gutierrez, 2021	105	20.952	13.621 to 29.986	37.67
Anne Rain T. Brown, 2020	20	5.000	0.127 to 24.873	17.22
Total (random effects)	366	14.057	7.451 to 22.330	100.00

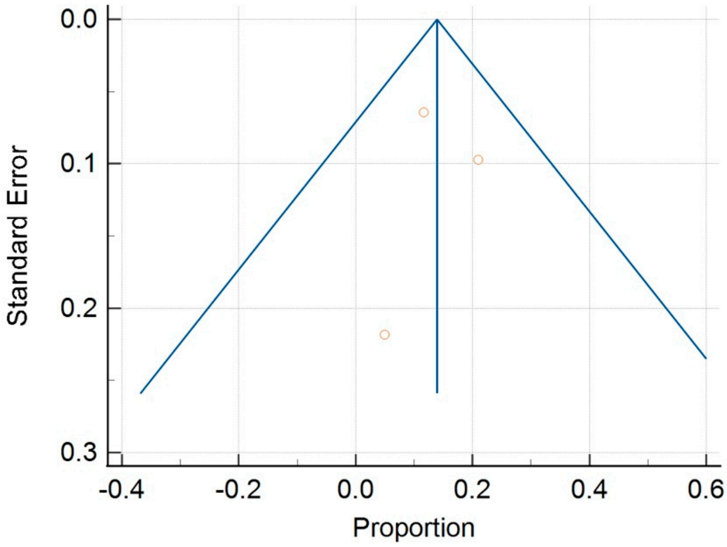

Test for heterogeneity

Q	6.0589
DF	2
Significance level	P = 0.0483
I^2 (inconsistency)	66.99%
95% CI for I^2	0.00 to 90.47

Publication bias

Egger's test	
Intercept	-0.1562
95% CI	-44.8011 to 44.4887
Significance level	P = 0.9717
Begg's test	
Kendall's Tau	0.3333
Significance level	P = 0.6015

Figure 19. Forest plot of proportions of CAR-T recipients who required high-flow nasal oxygen or noninvasive ventilation during their ICU stay, in published studies.

Figure 19 shows the combined proportion for our outcome of interest – patients who required high-flow nasal oxygen or noninvasive ventilation during their ICU stay, as a percentage. On average, 14% CI-95% [7,5-22] of the CAR-T recipients required high-flow nasal oxygen or noninvasive ventilation during their ICU stay. A substantial level of heterogeneity was reported (67%).

20. Vasoactive drugs

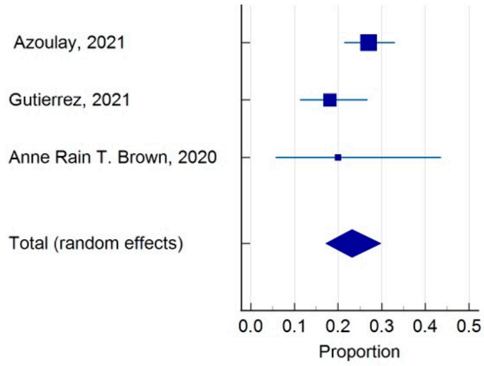

Study	Sample size	Proportion (%)	95% CI	Weight (%)
				Random
Azoulay, 2021	241	26.971	21.475 to 33.043	53.01
Gutierrez, 2021	105	18.095	11.261 to 26.807	36.07
Anne Rain T. Brown, 2020	20	20.000	5.733 to 43.661	10.92
Total (random effects)	366	23.188	17.122 to 29.870	100.00

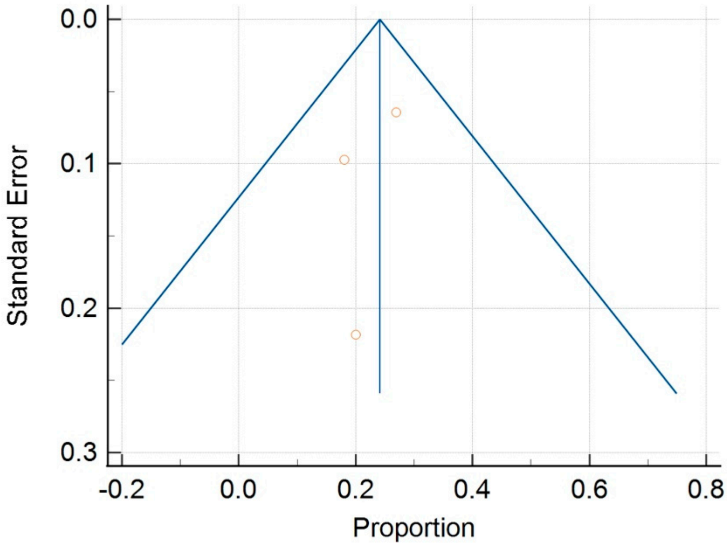

Test for heterogeneity

Q	3.2783
DF	2
Significance level	P = 0.1941
I^2 (inconsistency)	38.99%
95% CI for I^2	0.00 to 81.05

Publication bias

Egger's test	
Intercept	-1.4323
95% CI	-28.8071 to 25.9426
Significance level	P = 0.6265
Begg's test	
Kendall's Tau	-0.3333
Significance level	P = 0.6015

Figure 20. Forest plot of proportions of CAR-T recipients who required vasoactive drugs during ICU stay, in published studies.

Figure 20 shows the combined proportion for our outcome of interest – patients who required vasoactive drugs during ICU stay, as a percentage. On average, 23% CI-95% [17-29] of the CAR-T recipients required vasoactive drugs during their ICU stay. A moderate level of heterogeneity was reported (39%) with a p>0.10.

21.Renal replacement therapy

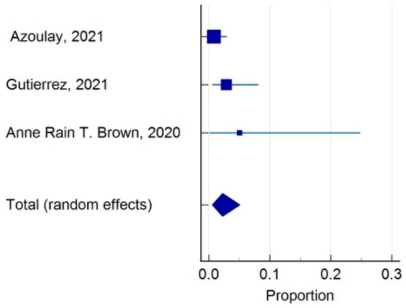

Study	Sample size	Proportion (%)	95% CI	Weight (%)
				Random
Azoulay, 2021	241	0.830	0.101 to 2.965	51.89
Gutierrez, 2021	105	2.857	0.593 to 8.123	36.49
Anne Rain T. Brown, 2020	20	5.000	0.127 to 24.873	11.63
Total (random effects)	366	2.236	0.503 to 5.162	100.00

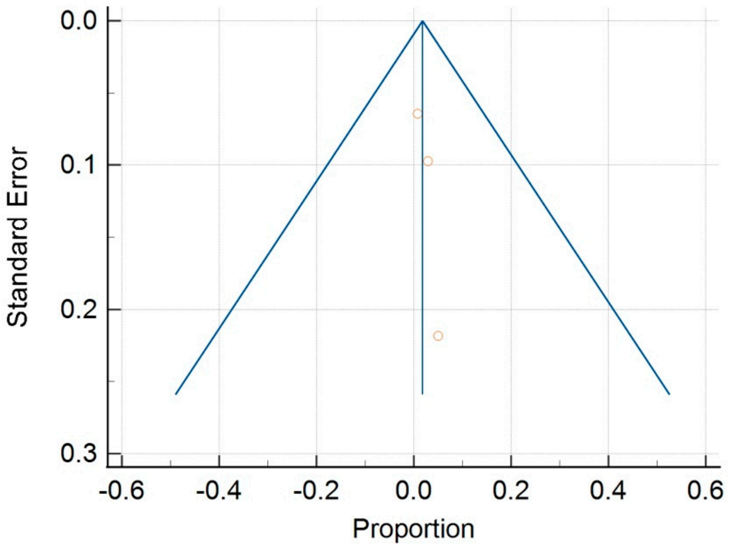

Test for heterogeneity

Q	3.5054
DF	2
Significance level	P = 0.1733
I^2 (inconsistency)	42.95%
95% CI for I^2	0.00 to 82.83

Publication bias

Egger's test	
Intercept	2.4404
95% CI	-11.4866 to 16.3673
Significance level	P = 0.2687
Begg's test	
Kendall's Tau	1.0000
Significance level	P = 0.1172

Figure 21. Forest plot of proportions of CAR-T recipients who required renal replacement therapy (RRT) during ICU stay, in published studies.

Figure 21 shows the combined proportion for our outcome of interest – patients who required RRT during ICU stay, as a percentage. On average, 2% CI-95% [0,5-5] of the CAR-T recipients required RRT during ICU stay. A moderate level of heterogeneity was reported (43%) with a p>0.10.

22.Mechanical ventilation during ICU stay

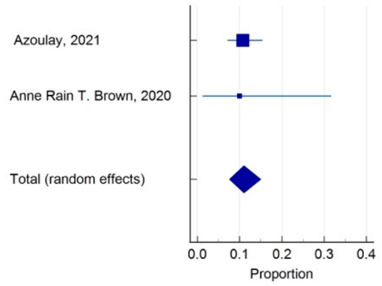

Study	Sample size	Proportion (%)	95% CI	Weight (%)
				Random
Azoulay, 2021	241	10.788	7.169 to 15.407	92.02
Anne Rain T. Brown, 2020	20	10.000	1.235 to 31.698	7.98
Total (random effects)	261	11.016	7.526 to 15.076	100.00

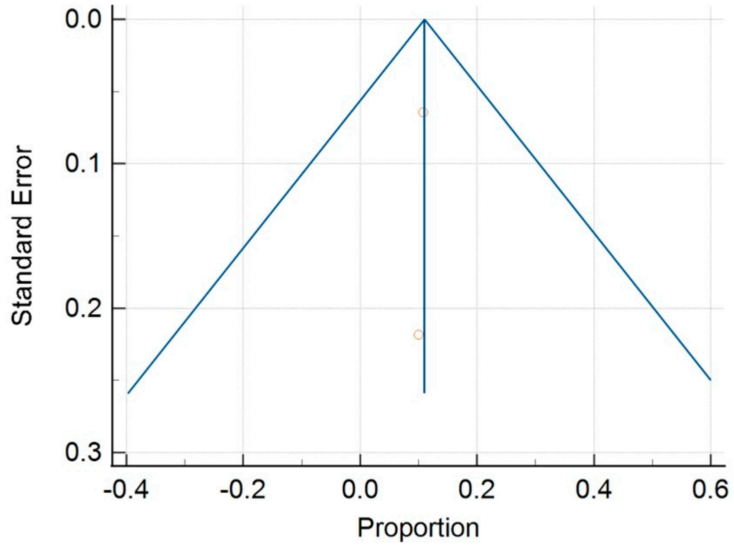

Test for heterogeneity

Q	0.01389
DF	1
Significance level	P = 0.9062
I^2 (inconsistency)	0.00%
95% CI for I^2	0.00 to 0.00

Publication bias

Egger's test	
Intercept	0.1741
95% CI	
Significance level	P < 0.0001
Begg's test	
Kendall's Tau	1.0000
Significance level	P = 0.3173

Figure 22. Forest plot of proportions of CAR-T recipients who required invasive mechanical ventilation during ICU stay, in published studies.

Figure 22 shows the combined proportion for our outcome of interest – patients who required invasive mechanical ventilation during ICU stay, as a percentage. On average, 11% CI-95%[7,5-15] of the CAR-T recipients required invasive mechanical ventilation during ICU stay. A 0% level of heterogeneity was reported with a p>0.10.

23.Readmission to the ICU within 30 days

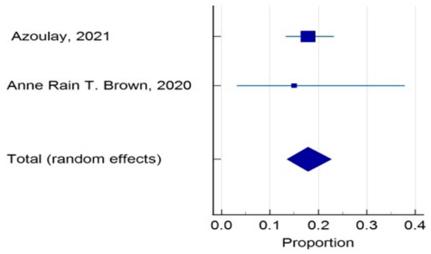

Study	Sample size	Proportion (%)	95% CI	Weight (%)
				Random
Azoulay, 2021	241	17.842	13.224 to 23.271	92.02
Anne Rain T. Brown, 2020	20	15.000	3.207 to 37.893	7.98
Total (random effects)	261	17.863	13.480 to 22.716	100.00

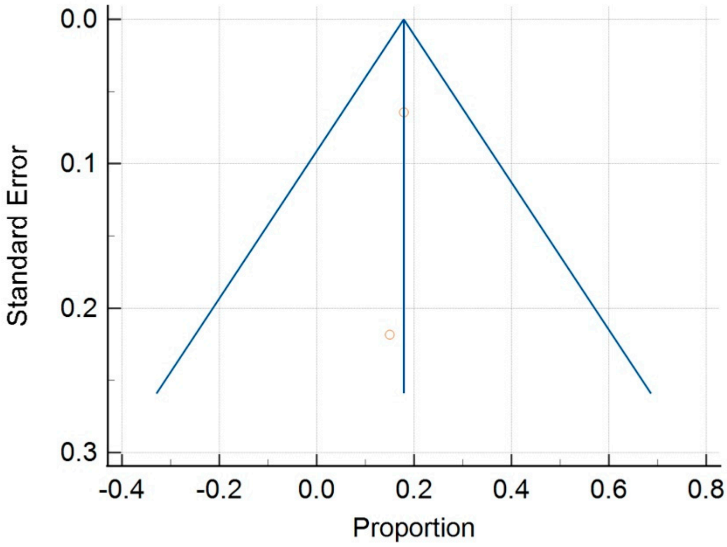

Test for heterogeneity

Q	0.02562
DF	1
Significance level	P = 0.8728
I^2 (inconsistency)	0.00%
95% CI for I^2	0.00 to 0.00

Publication bias

Egger's test	
Intercept	-0.2365
95% CI	
Significance level	P < 0.0001
Begg's test	
Kendall's Tau	-1.0000
Significance level	P = 0.3173

Figure 23. Forest plot of proportions of CAR-T recipients who were readmitted to the ICU within 30 days, in published studies.

Figure 23 shows the combined proportion for our outcome of interest – patients who were readmitted to the ICU within 30 days, as a percentage. On average, 18% CI-95% [13,5-23] of the CAR-T recipients were readmitted to the ICU within 30 days. A 0% level of heterogeneity was reported with a p>0.10.

24.Malignancy

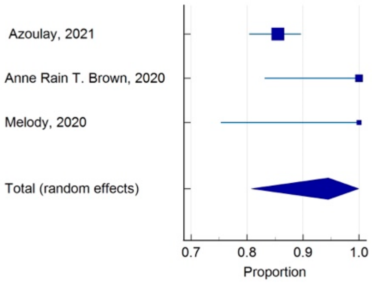

Study	Sample size	Proportion (%)	95% CI	Weight (%)
				Random
Azoulay, 2021	241	85.477	80.385 to 89.671	42.31
Anne Rain T. Brown, 2020	20	100.000	83.157 to 100.000	30.84
Melody, 2020	13	100.000	75.295 to 100.000	26.85
Total (random effects)	274	94.508	80.730 to 99.965	100.00

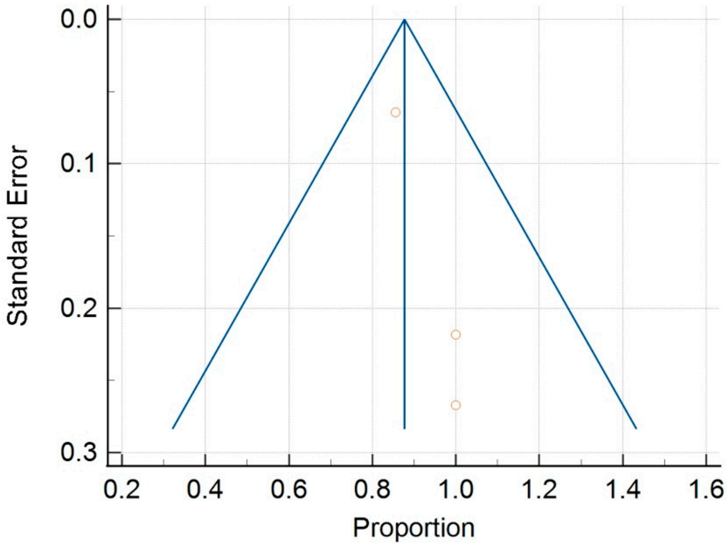

Test for heterogeneity

Q	9.1328
DF	2
Significance level	P = 0.0104
I^2 (inconsistency)	78.10%
95% CI for I^2	29.36 to 93.21

Publication bias

Egger's test	
Intercept	3.0556
95% CI	-4.6350 to 10.7462
Significance level	P = 0.1245
Begg's test	
Kendall's Tau	0.3333
Significance level	P = 0.6015

Figure 24. Forest plot of proportions of patients who had the diagnosis of lymphoma or follicular lymphoma for which they received CAR-T therapy, in published studies.

Figure 24 shows the combined proportion for our outcome of interest – patients who had a diagnosis of lymphoma or follicular lymphoma for which they received CAR-T therapy, as a percentage. On average, 95% CI-95%[80-99] of the CAR-T recipients had a diagnosis of lymphoma or follicular lymphoma. A considerable level of heterogeneity was reported (78%).
